# Supplementary material for: Sleep Promoting Effects of Lettuce (Lactuca sativa L.) Extracts in Korean Adults with Poor Sleep Quality: A Randomized, Double-Blind Placebo-Controlled Trial
Source: Nutrients. 2025 Jun 30;17(13):2172. doi: 10.3390/nu17132172 (PMC12251321; doi:10.3390/nu17132172)
Supplement: Supplementary file 1 [file nutrients-17-02172-s001.zip › Supplementary Tables_revised.pdf]

Supplementary Table S1. Within-group comparisons of subjective sleep quality measures before and after the intervention

	Test group (n = 47)		Placebo group (n = 47)	
	**Baseline**	**Final**	**Baseline**	**Final**
ISI	12.91 ± 3.55	6.96 ± 3.50 ***	12.00 ± 2.73	6.93 ± 3.36 ***
ESS	6.70 ± 2.75	4.89 ± 2.77 ***	6.00 ± 2.97	4.95 ± 2.80 *
SSS	2.70 ± 0.78	2.04 ± 0.62 ***	2.41 ± 0.82	2.23 ± 1.05
BDI	14.34 ± 6.85	9.66 ± 6.71 ***	14.09 ± 7.47	10.80 ± 7.62 ***
BAI	9.00 ± 5.79	5.17 ± 4.81 ***	8.66 ± 5.71	5.70 ± 5.58 ***
FSS	29.38 ± 10.93	24.02 ± 10.11 ***	25.91 ± 0.72	23.55 ± 11.07

ISI, Insomnia severity index; ESS, Epworth sleepiness scale; SSS, Stanford sleepiness scale; BDI, Beck depression inventory; BAI, Beck anxiety inventory; FSS, Fatigue severity scale.
Values are expressed as means ± SD.
Within-group differences from baseline to final assessed using paired t-tests: * $p < 0.05$, *** $p < 0.001$.

Supplementary Table S2. Within-group comparisons of vital signs before and after the intervention

	Test group (n = 47)		Placebo group (n = 47)	
	**Baseline**	**Final**	**Baseline**	**Final**
Systolic BP (mmHg)	116.20 ± 9.23	117.00 ± 10.95	119.20 ± 11.82	119.10 ± 10.07
Diastolic BP (mmHg)	75.64 ± 8.34	75.36 ± 7.77	77.12 ± 8.60	77.32 ± 7.31
PR (beat/min)	73.08 ± 10.02	75.30 ± 9.80	73.60 ± 8.81	74.40 ± 10.88

BP, blood pressure; PR, purse rate.
Values are expressed as means ± SD.
No significant within-group changes were observed in either group based on paired t-tests.

Supplementary Table S3. Within-group comparisons of laboratory test before and after the intervention

	Test group (n = 47)		Placebo group (n = 47)	
	Baseline	Final	Baseline	Final
WBC (10^3/μL)	5.42 ± 1.16	5.66 ± 1.31	5.59 ±1.50	5.57 ±1.39
RBC (10^6/μL)	4.33 ± 0.26	4.37 ±0.31	4.43 ± 0.37	4.43 ± 0.35
Hemoglobin (g/dL)	13.31 ± 1.04	13.37 ± 1.12	13.51 ±1.40	13.57 ± 1.28
Hematocrit (%)	39.87 ± 2.65	40.08 ± 2.97	40.57 ±3.54	40.36 ± 3.27
MCV (fL)	92.11 ± 5.04	91.93 ± 5.17	91.70 ± 5.43	91.17 ± 5.66**
MCH (pg)	30.74 ± 2.12	30.66 ± 2.13	30.51 ±2.49	30.64 ± 2.43
MCHC (g/dL)	33.36 ± 0.89	33.34 ± 1.07	33.24 ±1.28	33.58 ± 1.15**
PLT (10^3/μL)	237.40 ± 48.81	246.3 ±49.97*	249.10 ± 57.29	250.8 ± 55.24
Neutrophil (%)	52.76 ± 8.45	50.81 ± 9.19*	52.99 ±8.63	50.83 ± 10.34
Lymphocyte (%)	36.91 ± 7.40	38.83 ± 8.59*	36.19 ±8.57	38.16 ± 9.59
Monocyte (%)	5.22 ±1.19	5.20 ±1.35	5.24 ±0.94	5.37 ±1.02
Eosinophil (%)	2.44 ± 2.03	2.34 ±1.49	2.83 ± 2.43	2.82 ±2.30
Basophil (%)	0.51 ±0.27	0.58 ±0.26*	0.54 ±0.25	0.53 ± 0.29
ANC (cells/μL)	2894.3±908.6	2851.6±1065.8	3030.4±1128.6	2885.5±1069.9
Total Protein (g/dL)	7.25 ± 0.29	7.18 ±0.43	7.20 ± 0.41	7.17 ±0.42
Albumin (g/dL)	4.32 ±0.15	4.31 ±0.19	4.33 ±0.19	4.33 ±0.23
AST (U/L)	23.34 ± 6.36	24.83 ± 9.12	24.30 ±6.17	25.11 ± 6.98
ALT (U/L)	19.26 ± 7.75	21.23 ±3.56	21.81 ±9.50	23.21 ±0.17
Glucose (mg/dL)	101.50 ± 7.66	103.50 ±.88*	104.20 ±7.94	102.20 ±.10
BUN (mg/dL)	13.72 ± 3.49	13.64 ± 3.72	14.13 ±3.37	14.02 ± 3.93
Creatinine (mg/dL)	0.69 ±0.12	0.69 ±0.12	0.68 ± 0.13	0.67 ± 0.13

WBC: White blood cell; RBC: Red blood cell; MCV: Mean corpuscular volume; MCH: Mean corpuscular hemoglobin; MCHC: Mean corpuscular hemoglobin concentration; PLT: Platelet count: ANC: Absolute neutrophil count; AST: Aspartate aminotransferase; ALT: Alanine aminotransferase; BUN: Blood urea nitrogen

Values are expressed as means ± SD.

Within-group differences from baseline to final assessed using paired t-tests: * $p < 0.05$, ** $p < 0.01$.
